# Application of a Non-Linear multi-model Ayurveda Intervention in elderly COVID-19 patients- a retrospective case series

**DOI:** 10.1016/j.jaim.2021.06.016

**Published:** 2021-07-02

**Authors:** K.S. Dinesh, P.K. Nazeema, Madhavi Archana, K. Jayakrishnan, A.S. Santhi Krishna, S. Swapna Chitra, V.K. Sujitha, Anju Sathian, M. Girish Babu, Geethu Balakrishnan, C. Krishnendhu

**Affiliations:** a Department of Kaumarabhritya, Vaidyaratnam P S Varier Ayurveda College, Kottakkal. Edarikode (P.O), 676501, Malappuram District, Kerala, India; b AYUSH Public Health Initiative, Department of Kaumarabhritya, Vaidyaratnam P S Varier Ayurveda College, Kottakkal Edarikode (P.O), 676501, Malappuram District, Kerala, India; c Department of Swasthavritha, All India Institute of Ayurveda, Mathura Rd, Gautampuri Awas, Sarita Vihar, New Delhi, 110076, India; d Surya, Katampazhipuram, Palakkad, 678633, Kerala, India; e Department of Kaumarabhritya, Santhigiri Ayurveda Medical College, Olassery, Kodumba, Palakkad, 678551, Kerala, India; f Department of Statistics, CHMKM Government Arts and Science College, Koduvally, 673572, Kozhikode, India; g AYUSH Extra Mural Research, Department of Kaumarabhritya, Vaidyaratnam P S Varier Ayurveda College, Kottakkal, Edarikode (P.O), 676501, Malappuram District, Kerala, India

**Keywords:** Ayurveda, COVID-19, Elderly patients, Alternative medicine, Co-morbidity, Traditional medicine, Survival function, SARS-CoV-2, Severe Acute Respiratory Syndrome Corona Virus-2, COVID-19, Corona Virus Disease- 2019, CAM, Complementary and alternative medicine, NRIs, Non-Resident Indians, RT-PCR, Reverse Transcription-Polymerase Chain Reaction, NLMAI, Non-Linear multi-modal Ayurveda Intervention, AYUSH, Ayurveda, yoga and naturopathy, Unani, Siddha and Homeopathy

## Abstract

The COVID-19 pandemic is ravaging the world, leaving the mainstream medical system handicapped with no proven treatment at one end and the ambiguities regarding the efficacies of vaccines at the other. The elderly population is at greater risk in terms of complications and death. The use of complementary and alternative medicine (CAM) against COVID-19 has already been documented in countries like China with a national participation rate of 90%. In this regard, the practice of CAM especially Ayurveda is relevant in India. The current report is a case series of 64 elderly COVID-19 patients managed through a Non-Linear multi-modal Ayurveda Intervention (NLMAI) via online consultation. NLMAI is a combination of herbal and herbo-mineral drug interventions, lifestyle modifications, and psychological support done in 2 phases. The post-management analysis revealed a mean duration of 11 symptoms of COVID-19 assessed through survival function as 0.577 days [SE=0.39] with a CI of 95% [0.500–0.653] which was considerably low when compared to global statistics. Moreover, none of the cases advanced to complications or death. Hence, novel approaches like NLMAI can be utilized to counter the gravity of the COVID-19 after scientific validation.

## Graphical abstract

**Figure:**
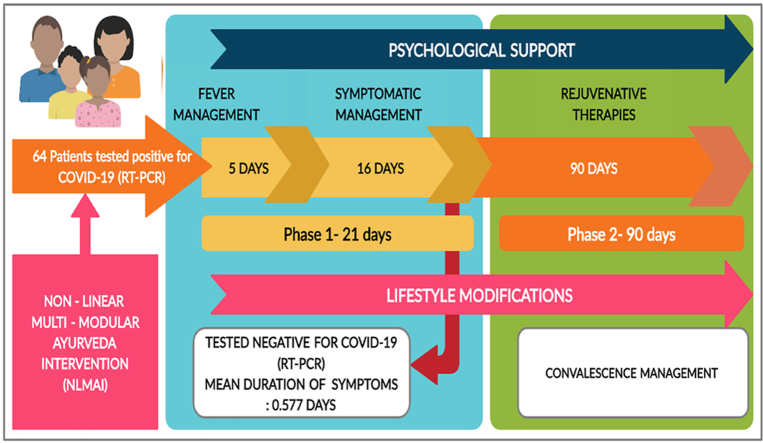

## 1 Introduction

The Severe Acute Respiratory Syndrome Corona Virus-2 (SARS-CoV-2) causing the Corona Virus Disease (COVID-19) was first identified in Wuhan, Hubei province of Peoples Republic of China.With over 155 million infections, nearly 32 lakh deaths, and an economic toll accounting to trillions, the COVID-19 pandemic is ravaging the world [1]. The global health workforce fighting against COVID-19 and other infirmities is limited to 4.45 doctors, nurses and midwives per 1000 population indicating a low health worker density [2]. The health worker density is even sparser in india with only 2.09 health workers per 1000 population of which 22.8% are AYUSH (Ayurveda, yoga and naturopathy, Unani, Siddha and homoeopathy) practitioners [3] facilitating the propagation of these systems of medicine in the society especially in Kerala, a state of India. Furthermore, Kerala gained global reputation, in containing the exponential rise of COVID-19 cases through rigorous containment strategies some months back. However, the current test positivity rate has shoot past 25% on average but with a minimal fatality rate of 0.4% [4].

The Non-Residential Indians (NRI's) of severely infected countries of the Middle East, with limited access to health services due to national priority concerns of host nations and economic stringencies, sought health care measures of traditional medicinal system of Ayurveda. The conventional medical system had the challenge of patient management with no proven treatment for COVID-19, which was a rising public concern. The current report is a retrospective case series of 64 Non-Resident Indians (NRIs) 60 years or older tested positive for COVID-19 sorted treatment due to apprehension towards modern medicinal system and inclination towards indigenous systems, managed through the Non-Linear multi -modal Ayurveda Intervention (NLMAI) for 21 days.

## 2 Patient information

The report includes a multicentered retrospective case series of 64 NRI patients above 60 years of age, among 300 COVID-19 patients from 9 Middle East nations, who voluntarily sought Ayurveda mode of treatment between the period of 23rd of March 2020 and the 26th of June 2020 through various online media. The demographic, domiciliary, clinical data and exposure history were recorded at the time of consultation. The major clinical presentations were fever, cough, dyspnea, headache, fatigue, myalgia, dizziness, nasal congestion, rhinorrhea and abnormal digestion. Along with that, co-morbidities were also reported. Assessed co-morbidities were obesity, hypertension, Diabetes mellitus, cardiovascular disease and COPD. The cases were reluctant to take any sort of Conventional antiviral or antibiotic therapies, and preffered to sought Covid management through Ayurveda at its preliminary stage following diagnosis. The patients were categorized into two groups according to the Ayurveda epistemological approach.

## 3 Demographic findings

The reported cases were 64 elder citizens (Male/Female = 45/19) above 60 years of age with a mean age of 66.4 years. On tracing the domiciliary status, majority of the identified cases underwent room isolation (95.3%) in their own houses and the rest were isolated in hotels. The subjects reported positive exposure from workplace (50%) and through domiciliary close contacts (50%), whereas no one reported exposure due to international travel (Table 1). The diagnosed co-morbidities are given in Table 2.

**Table-1:** Demographic profile of the patients.

Sl No	Variables			No of cases (%)
1	Demographic Characteristics	Age groups	60–65	26 (40.6)
			66–70	35 (54.6)
			Above 70	3 (4.7)
			Total	64
		Gender	Male	45 (70.3)
			Female	19 (29.7)
2	Domiciliary Status	Hospital		0(0)
		Room		61 (95.3)
		Hotel		3 (4.7)
3	Exposure History	Travel		0(0)
		Workplace		32 (50)
		Close contact		32 (50)

**Table 2:** Percentage of Comorbidities.

Co-morbidities	Frequency	Percent
Obesity	12	19
Hypertension	30	47
Diabetes Mellitus	28	44
Cardiovascular disease	18	28
COPD	5	8
HIV, Malignancy, Renal disorders, Immuno-deficiency state	Nil	0

## 4 Timeline (Fig. 1 Timeline of the study)

The first patient was recruited on 23^rd^ March 2020, each patient went through initial symptomatic management and 3 follow-ups were done (every seventh day). After this symptomatic management of 21 days, a phase 2 rejuvenative therapy was administered for 2 months. Lifestyle modifications and psychological support were ensured among the patients throughout the whole interventional period. Patient recruitment continued up to 26^th^ June 2020.

**Fig. 1:**
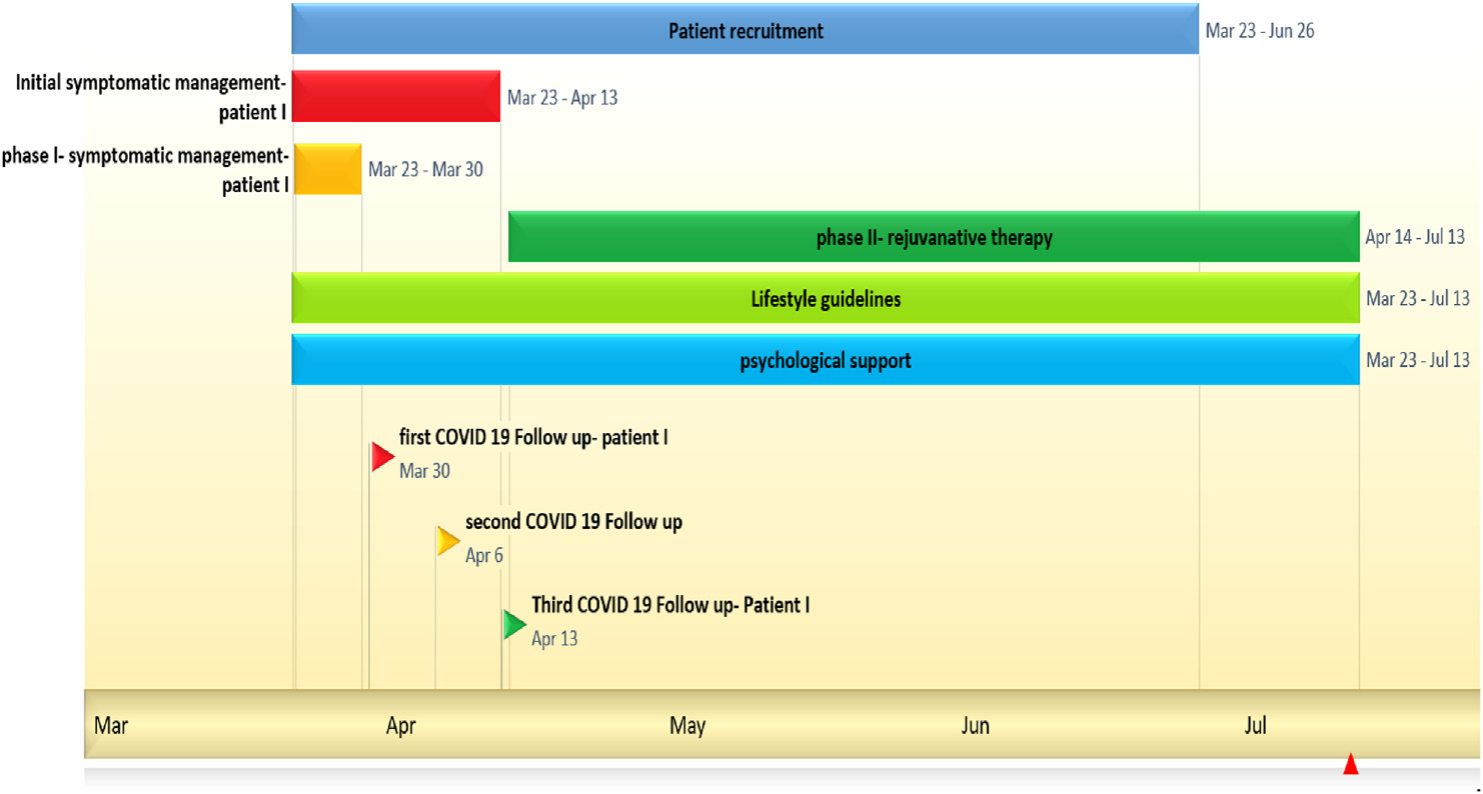
Timeline.

## 5 Diagnostic assessment

The included cases were diagnosed for COVID-19 using the RT-PCR tests according to the guidelines on treatment and prevention laid down by the health ministries of the respective countries and previously diagnosed with co-morbidities. All the diagnosed cases were under isolation and followed Ayurveda interventions as per their personal choice. The clinical symptoms of the patients were recorded by the consultant doctor of Ayurveda, analyzed and periodic follow up was made through online consultation. An Ayurveda mode of diagnosis is also parallelly done for the accurate assessment and delivery of therapeutics as it provides a personalized and preventive health care [5].

## 6 Therapeutic intervention

On the ground of the epistemological foundation of Ayurveda, the patients were assigned into two intervention groups (Groups A&B)^1^ considering the presenting symptom complex in accordance with the predominant *dosha* of the associated symptoms [The patho-physiological background for clinical assessment and therapeutics] [6]. (Table .3) The main aim of interventions was to manage the symptoms of COVID-19 (Phase-1), reduce the possible complications due to co-morbidities in elderly and subsequently improve the immunity of the convalescing patients (Phase-2). The interventions were of nonlinear, dynamic and complex in nature [7]. Thees included administration of polyherbal or herbo-mineral compounds (as per the Ayurveda Pharmacopeia of India) which are commonly used for *jwara, swasa* and *kasa* (fever and respiratory ailments), lifestyle guidelines and psychological support. It demands individual customization justifying Ayurveda epistemology and availability of the drugs in respective nations [5]. This approach was collectively termed as Non-Linear Multi-modal Ayurveda Intervention (NLMAI). However, no Ayurveda medication was prescribed for co-morbidities and the concomitant medications were advised to be continued for the same.

**Table 3:** Administration of NLMAI.

	GROUP A (*Kapha* predominant)		GROUP B (*Pitta* predominant)
Medicines	Fever Management	*Vettumaran Gutika*^a^ *Septillin* Tablet^b^	*Sudarshanam Gutika*^a^ *Shadanga Paneeyam*^a^ *Septillin* Tablet
	General immune boosters	*RajanyadiChurnam*^a^ *AvipathiChurnam*^a^ *Haridrakhanda*^a^	
	Conditional medications	*Dasamoola Katuthrayam Kashayam*^a^	*Dadimadhi choornam*^a^ *Vilwadi Gutika*^a^ *Tulsi* steam inhalation
Dose and Duration	As per standard age-based Posology– for 21 days		As per standard age-based Posology– for 21 days
Other Interventions and Duration	Lifestyle guidelines - Psychologically active interrogative sessions -		Lifestyle guidelines - Psychologically active interrogative sessions -
Follow up medicines & Duration	*Rasayana* concept medicines – 3 months *Agastya Rasayana*^a^		*Rasayana* concept medicines – 3 months *Chyavanaprasha*^a^

a Ayurveda Pharmacopeia of India, Pharmacopoeia Commission for Indian Medicine & Homoeopathy.

b Patent and Proprietary Ayurvedic Medicine.

### 6.1 Lifestyle guidelines in NLMAI

• Disciplined observance of routines like sleep–wake cycle, toileting, exercising, bathing and dining for maintaining a healthy biological clock.
• Luke warm water for shower (contraindicated during fever).
• Steam inhalation with leaves of basil (*Ocimum sanctum*) daily.
• Strict observance of self-isolation, universal masking and handwashing as part of mitigation measures.
• Avoid exposure to anything that cause mental stress (sensational news, social media posts and the like)

### 6.2 Diet modifications in NLMAI

• Observance of healthy food and eating practices as per Ayurveda
• Follow strict vegetarian diet.
• Intake of Herbal drinks/decoctions processed with *O. sanctum* leaves and *Zingiber officinale* tubers - twice a day.
• Avoid intake of too much sour, salty and spicy food items.
• Light diet with plenty of liquids preferably rice gruel.
• Avoid refrigerated foods, aerated drinks, junk foods, picky eating and confectionaries.

The prescriptions were generated and sent after the online consultation. The patients were asked to collect the medicines according to the legal norms of the countrries they were residing. The contact details of voluntary services, human welfare organizations and proximate Ayurveda practitioners were shared to them who could help in dispensing the medicines.

## 7 Follow-up and outcomes

The patients were requested to invariably report to the doctors through available online/telephonic media at regular intervals (at least once in 7 days from the initial consultation) or at any time in case of emergencies. The outcomes were recorded for both phase-1 and phase-2 treatments based on patient self-reporting and clinician's interrogations. Even though the rejuvenative (*Rasayana*) drugs were prescribed in second phase of the treatment, the assessment in the present study was focused only to phase-1 treatment and co-morbidities. The Patient adherence and compliance to the interventions was ensured through ‘telephone-based pill count method’ and periodic phone calls. The adherence to the lifestyle guidelines was ensured through the online family interviews. The Psychological support to the elderly was also ensured adopting the guidelines of government supported program “*Koode*” organized by the Malappuram district governance of Kerala state. The data was collected during online consultations and follow-up records were stored in excel sheets. Informed consent from each patient was procured verbally prior to study recruitment.

The patients were assessed based on commonly reported clinical symptoms of COVID-19 as per authentic online database and were subjected to assessment. Among them, sore throat (100%), fever (92%), anosmia (28%), nasal congestion (20%), cough (19%), Rhinorrhea (19%), myalgia (19%) and fatigue (13%) and were most prevalent, followed by, Diarrhea (6%), Abnormal digestion (5%) and Dizziness (3%) (Table 4). For the ease of therapeutic intervention, the patients were categorized into two groups based on the associated symptoms. Hyperemia due to inflammation of throat, mouth and nose, skin rashes, burning sensations and diarrhea were included in *pitta* category and Rhinitis, chills, feverishness, heaviness and loss of appetite were categorized under *kapha*.

**Table 4:** Symptom complex and its incidence.

SI No	Symptom complex	Symptom Prevalence (%)
1	Sore throat	100
2	Fever	92
3	Anosmia	28
4	Nasal Congestion	20
5	Cough	19
6	Rhinorrhea	19
7	Myalgia	19
8	Fatigue	13
9	Diarrhea	6
10	Abnormal digestion	5
11	Dizziness	3

**Fig. 2:**
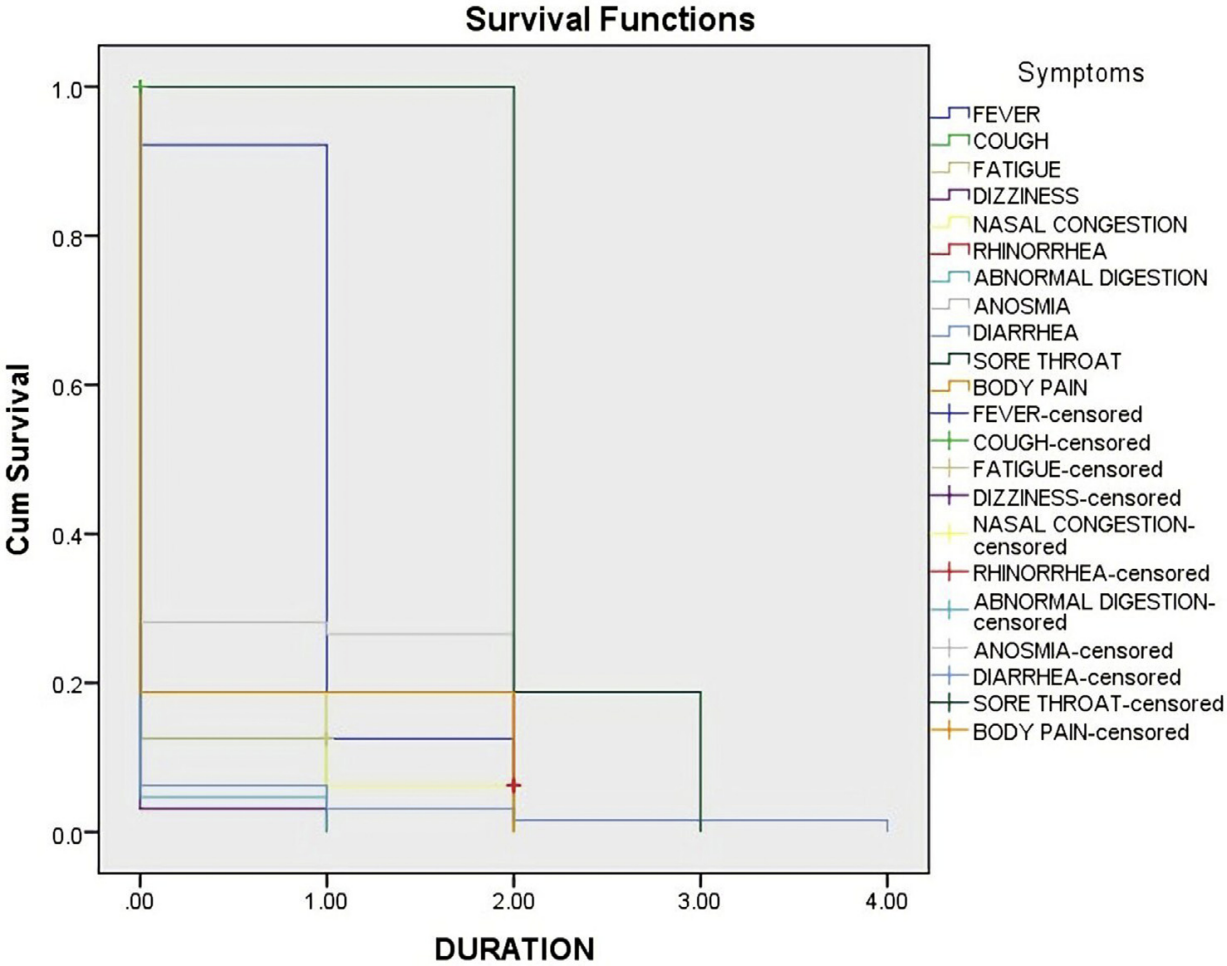
Duration of COVID-19 clinical symptoms.

The overall duration of symptoms was 0.58 days [SE = .39] with a CI 95% [lower bound = 0.500, upper bound = 0.653] (Table 5), (Fig. 2 The overall duration of symptoms) indicating speedy recovery when managed through NLMAI. In the present report, no adverse situations or fatalities were reported during the observation period.

**Table 5:** The mean duration(days) of symptoms after NLMAI.

Symptoms	Mean duration (days)			
	Estimate	Std. Error	95% Confidence Interval	
			Lower Bound	Upper Bound
Fever	1.172	0.091	0.994	1.349
Cough	0.125	0.042	0.043	0.207
Fatigue	0.125	0.041	0.044	0.206
Dizziness	0.031	0.022	0.000	0.074
Nasal congestion	0.313	0.097	0.123	0.502
Rhinorrhea	0.688	0.186	0.323	1.052
Abnormal digestion	0.047	0.027	0.000	0.099
Anosmia	0.844	0.176	0.499	1.188
Diarrhea	0.172	0.088	0.000	0.344
Sore throat	2.188	0.049	2.091	2.284
Body pain	0.375	0.098	0.182	0.568
**Overall**	0**.577**	**0.039**	**0.500**	**0.653**

## 8 Discussion

The year 2020 has engraved its signature in the history of mankind as it led to a situation of human isolation, fear and stagnation not known for a century. The COVID-19 pandemic swept through continents creating havocs to mankind in almost all countries irrespective of developments in technology and medicine. India with its high population density, is hit badly by the pandemic. This has forewarned the heterogenous medical structure of India comprising both conventional and the AYUSH systems. In the initial phases of the infection, the health ministry endorsed conventional system for the treatment of COVID-19 and the AYUSH systems to be used for preventive strategies [8]. Permissions were denied for AYUSH doctors to treat COVID-19 cases ignoring the examples set by the Chinese government which permitted TCM practitioners to treat COVID-19 cases with national participation rate of 90% and thereby proved it significantly effective in prophylaxis than the conventional system [9]. A study astonishingly revealed that, out of the 60,107 cases treated by TCM, variables like clinical symptoms' disappearance time, the recovery time of body temperature, the average length of stay in hospital, the time of nucleic acid turning negative was shortened significantly [10]. Such an intervention was made possible by the integration of TCM in the Chinese National Health Policy. However, the Government of India has initiated the ‘Interdisciplinary AYUSH Research and Development Task Force’ with the objectives of reducing the suffering and deaths associated with COVID-19 in India through grants for active clinical trials, prevention strategies, immunity enhancement programs and convalescence management [11].

Meanwhile, physicians of Ayurveda through mainstream journals have discussed pragmatic plans for the management of COVID-19 [12]. But the denial of legal validation for physicians of alternative medical systems to execute such plans have crippled their spirits during this phase of disaster. There exists a perplexity among general public regarding the COVID-19, its spread and management due to its novelty. This has created a dilemma in choosing which system of medicine to abide with, the hospital or the physician to consult.

Kerala, a state of India unlike the others, strategically managed the initial pandemic phase by an active participation of 2.36 Lakhs volunteers (*Samoohya sannadha sena*) from the general public [13]. This facilitated the promotion of intrinsic traditional health practices and an unreserved affinity of Indian people towards the role of traditional health systems like Ayurveda in COVID-19 [14]. In addition, the impression that, the mainstream medical system had no proven treatment for COVID-19 and prevailing ambiguities on its efficacy and complications of the management protocols might have led the patients to prioritize Ayurveda over the conventional therapies, leading them (64 cases from the present study) to approach a team of doctors of a reputed Ayurveda institution of Kerala.

Majority of the NRIs from varios socio-economic stratas, currently based in the middle east left the home country seeking different jobs [15]. Thus, Among the reported 300 patients for treatment through NLMAI, 64 elderly patients with a mean age of 66.4 years with co-morbidities were included in the analysis. Majority of the participants were men (70.3%). They reported absence of hospital visits in the meantime due to fear of nosocomial infections and secondary reactions. It was also noted that 4.7% cases preferred hotel quarantine and denied hospital quarantine due to the fear of complications and related mental health disturbances like depression [16]. The reported elderly patients were not prescribed steroids, antibiotics, blood thinners and/or Remdesivir through dextrose saline in pneumonia. Thus, drug induced catastrophes were not reported.

The housing of the expats ranged from labour camps, small peer groups, families or by themselves. The cases reported a history of close contact with their families, friends or with colleagues. It was evident that, the infections were a result of constant or multiple exposures compared to a single exposure. A relatively lower spread of infections to the family memebers of the infected persons, was probably due to preventive measures and lifestyle guidelines of NLMAI adopted by the family members and a better personal care.

Based on the previously reported data, the mean time period from onset of symptoms to its resolution was 8 days [17] (6.5–11.5). It must be noted that, the patients who underwent the NLMAI intervention revealed a much lower value (0.58 days [SE = .39] with a CI 95% [lower bound = 0.50, upper bound .653]). The drugs selected for the protocol aimed at fever management, general immunity boosting and pacifying the predominant *dosha* and it's associated symptoms in each group (Table 3). These are routine medicines used for the clinical management of *jwara*, *swasa* and *kasa,* their pharmacological actions are described detailed in Table 6.

**Table 6:** Mode of action of drugs.

Name of the drug	Dosage form	Pharmacological action	Reference
Vettumaran gulika	Tablet	CNS depressant	Ravishankar B, Sasikala CK. Pharmacological evaluation of compound Ayurvedic preparations: Part C: Vettumaran Gutika (VTG). Anc Sci Life. 1983; 3(1):11–18.
Septillin tab	Tablet	Tonsillitis, Pharyngitis, Laryngitis, Sinusitis, Rhinitis, Persistent cough, Common cold, Bronchitis, Immuno modulator.	Clinical Efficacy and Safety of Septilin Tablets in Respiratory Tract Infections: A Meta-analysis MedhaKshirsagar, D Palaniyamma, S Gopumadhavan, Pralhad S Patki Indian Journal of Clinical Practice, Vol. 20, No. 8, January 2010
Sudarsanam gulika	Tablet	Enlargement of liver and spleen, Fever, Intermittent fever, Chronic fever, Abdominal lump	AFI part 1, pgno.116
Shadanga paneeyam	Decoction	Thirst, Fever.	AFI part 1, pgno.200-201
Rajanyadi churnam	Powder	Diarrhea, Malabsorption syndrome, Jaundice, Anemia, Fever, Digestive impairment, Distension of abdomen due to obstruction to passage of urine and stools, Dyspnea/Asthma, Cough, Diseases of children, Weakness, Discolouration	AFI part 1, pgno.343-344 Deshpande R, Shreedhara CS, Setty Aswatha Ram HH. Standardization of Rajanyādi cūrṇa: An ayurvedic preparation. Anc Sci Life. 2014 Jan; 33(3):146–50. Doi: 10.4103/0257–7941.144617. PMID: 25,538,348; PMCID: PMC4264301.
Avipathy churnam	Powder	Digestive impairment, Constipation, Dyspepsia, Hemorrhoids, Retention of urine, Urinary disorders	AFI part 1, pgno.309-310
Haridrakhandam	Powder	Urticaria, Itching, Blister, Taeniasis, Urticaria.	AFI part 1, pgno.48
Dasamoola katutrayam kashayam	Decoction	Dyspnea/Asthma, Cough, Intercostal neuralgia and pleurodynia, Disease due to vata dosha, Pain in sacral region, Pain in the lower back, Headache.	AFI part 1, pgno. 174-175 Hariharan S Dr., Premvel SD Dr.; From 5th World Ayurveda Congress 2012 Bhopal, Madhya Pradesh, India. 7–10 Dec 2012. PA01.09. Efficacy of ayurvedic formulations in allergic asthma patients with special reference to elevation of TIgE. Anc Sci Life. 2012; 32(Suppl 1):S58.
Dadimadi churnam	Powder	Malabsorption syndrome	AFI part 1, pgno.117
Vilwadi gulika	Tablet	Poison of Scorpion, Spider and Snake Gastro-enteritis with piercing pain, Dyspepsia Effects of slow/accumulated poison, Fever, Psychological disorder.	AFI part 1, pgno.523-524 C T S, M D, P R R, K M, E M A, Balachandran I. Chemical profiling of selected Ayurveda formulations recommended for COVID-19. Beni Suef Univ J Basic Appl Sci. 2021; 10(1):2. Doi:10.1186/s43088-020-00089-1
Agastya rasayana	Electuary	Hiccough, cough, dyspnea, phthisis, intermittent fever, adapto-immuno-neuro-endocrino modulator	AFI part 1 Pgno. 111-112 Adluri USP, Tripathi AC. Understanding COVID - 19 pandemic - A comprehensive Ayurvedic perspective [published online ahead of print, 2020 Sep 8]. J Ayurveda Integr Med. 2020; S0975-9476(20)30,064–4. Doi:10.1016/j.jaim.2020.08.001 Warrier PR. Problems of aging jara cikitsa - the ayurvedic treatment for preventing and curing senility. Anc Sci Life. 1982; 1(4):210–215.
Chyavanaprasam	Electuary	Cough, dyspnea, debility due to chest injury, heart disease, adapto-immuno-neuro-endocrino modulator	AFI part 1 Pgno. 127-130 Sharma R, Martins N, Kuca K et al. Chyawanprash: A Traditional Indian Bioactive Health Supplement. Biomolecules. 2019; 9(5):161. Published 2019 Apr 26. Doi:10.3390/biom9050161
*Ocimum sanctum*. Linn	Single drug, **fresh leaves (**1 handful (∼10gms) in 1000ml of water)		Mondal S, Varma S, Bamola VD, Naik SN, Mirdha BR, Padhi MM, Mehta N, Mahapatra SC. Double-blinded randomized controlled trial for immunomodulatory effects of Tulsi (*Ocimum sanctum* Linn.) leaf extract on healthy volunteers. J Ethnopharmacol. 2011 Jul 14; 136(3):452–6. Doi: 10.1016/j.jep.2011.05.012. Epub 2011 May 17. PMID: 21,619,917.
*Zingiber officinale*	Single drug, **Dried tuber**, (5gms of crushed dried tuber in 1000ml of water)		Chang JS, Wang KC, Yeh CF, Shieh DE, Chiang LC. Fresh ginger (*Zingiber officinale*) has anti-viral activity against human respiratory syncytial virus in human respiratory tract cell lines. J Ethnopharmacol. 2013 Jan 9; 145(1):146–51. Doi: 10.1016/j.jep.2012.10.043. Epub 2012 Nov 1. PMID: 23123794.

Similarly, the time duration from virus positivity to negativity was 5.5 days (lower bound-4, upper bound-8). The present report could not draw an inference regarding this factor, as the patients were retested for SARS-nCoV-2 only on the 7th or 14th day in accordance with the goverment policies. Due to this, earlier clinical improvement compared to mean may not have reported. Thus, a planned prospective study is required to deduce the effectiveness of NLMAI in terms of time for negativity of COVID-19. A comparative clinical trial on the efficacy of NLMAI between the groups and with contemporary protocols can also be a research option.

In recent covid related studies, the median duration of fever and associated symptoms was 10 days (CI 95%; 8–11 days) [18]. In the present study, none of the participants received any hospital-based interventions. Yet, the average time for clinical improvement of fever and associated symptoms through NLMAI is found to be much less (0.577 days) (Table 5).

In addition, the reported co-morbidities were obesity (18.75%), hypertension (46.87%), diabetes mellitus (43.75%), cardiovascular disease (28.15%) and COPD (7.81%). COPD, Cognitive impairment, diabetes, hypertension, and stroke are significant contributions of health care utilization and hospital admissions among covid 19 patients [19]. Increased risk of mortality with advanced age were also reported [20,21] All available evidence suggest that presence of co-morbidities is associated with poor outcome of covid 19 patients [22,23] However, in the current study, no complications were reported in any of the affected cases during their course of illness and follow-up.

Individuals with above 65 years of age account for 4.5–11.2% of all COVID-19 deaths in European countries and Canada, it is 8.3–22.7% in the US, and were the majority in India and Mexico. People below 65 years of age have lower risks of COVID-19 death even in pandemic epicenters. Data revealed that, in India COVID-19 mortalities among the age group below 65 years were 49.5% of the total deaths and risk of death in people with age below 60 is 5 per million [24]. In the present case report, not even a single fatality due to COVID-19 was reported during the NLMAI treatment and follow-up period, which can be speculated as a beneficial outcome.

## 9 Conclusion

Among the 64 NRI elderly COVID-19 patients, NLMAI revealed a mean duration by assessing the survival function of 11 symptoms of COVID-19 as 0.577 days [SE = .39] with a CI 95% [lower bound = 0.500, upper bound 0.653] which was considerably low when compared to global statistics [10 days (CI 95%; (CI's: 8–11 days)]. Moreover, none of the cases with co-morbidities progressed to severe symptoms, complications or death during the period of intervention. All cases tested viral negativity on the 14th day as per the norms of their respective host countries.

## Informed consent

Informed consent was obtained from the patients through recorded telephonic conversations prior to study recruitment.

## Funding

International federation for Ayurveda-for preparation of article, analysis and interpretation of data.

## Conflict of interest

None.

## Author contributions

**K.S. Dinesh**: Conceptualization, Methodology, Validation, Resources, Data Curation, Writing - Review & Editing, Project administration and Funding acquisition.

**P.K. Nazeema:** Conceptualization, Methodology and Supervision.

**Madhavi Archana:** Writing **-** Original Draft, Writing - Review & Editing and Visualization.

**K. Jayakrishnan**: Writing - Original Draft, Writing - Review & Editing and Visualization.

**A.S. Santhikrishna**: Writing - Original Draft and Investigation.

**S. Swapna Chitra**: Investigation.

**V.K. Sujitha**: Investigation.

**Anju Sathian**: Investigation.

**M. Girish Babu**: Formal analysis and Data Curation.

**Geethu Balakrishnan:** Investigation.

**C. Krishnendu:** Investigation.

## Footnotes

1 The subjects were divided into group A (with *Kapha* predominant symptoms) and Group B (with *Pitta* predominant symptoms).
